# Global Ageing and Dementia Study Explorer (GLADSE)

**DOI:** 10.1002/alz70860_100011

**Published:** 2025-12-23

**Authors:** Rory Chen, Vibeke S. Catts, Ashleigh S. Vella, Sarah Bauermeister, Joshua Bauermeister, Simon Young, Caitlin McHugh, Mukta Phatak, Graham Bearden, John Gallacher, Matthew Clement, Perminder S. Sachdev

**Affiliations:** ^1^ Centre for Healthy Brain Ageing (CHeBA), UNSW Sydney, Sydney, NSW, Australia; ^2^ Dementias Platform UK ‐ University of Oxford, Oxford, United Kingdom; ^3^ Dementias Platform UK, Department of Psychiatry, University of Oxford, Warneford Hospital, Oxford, Oxfordshire, United Kingdom; ^4^ University of Oxford, Oxford, United Kingdom; ^5^ Alzheimer's Disease Data Initiative, Kirkland, WA, USA; ^6^ Alzheimer's Disease Data Initiative, Seattle, WA, USA; ^7^ Alzheimer's Disease Data Initiative, Kirkland, WA, USA; ^8^ University of Oxford, Oxford, Oxfordshire, United Kingdom; ^9^ Alzheimer's Disease Data Initiative (ADDI), Kirkland, WA, USA; ^10^ Neuropsychiatric Institute, Prince of Wales Hospital, Randwick, NSW, Australia

## Abstract

**Background:**

Data repositories are critical to advancing open science, ensuring data persistence, and promoting reuse for novel scientific discoveries. In dementia research, platforms such as Dementias Platform Australia, Dementias Platform UK, and the Alzheimer's Disease Data Initiative, have emphasized making data Findable, Accessible, Interoperable, and Reusable (FAIR). However, the decentralized nature of these platforms requires researchers to navigate multiple sites, compile metadata manually, and face challenges in comparing and accessing studies across platforms.

**Method:**

To address these challenges, we developed the Global Ageing and Dementia Study Explorer (GLADSE), an open‐source R Shiny application. The tool collects and standardizes commonly used metadata from partner platforms, including applying the C‐SURV data model themes and domains, to provide rich, detailed and comparable information. The application was built using R Shiny modules under the *golem* framework, ensuring modularity for scalability and ease of maintenance. GLADSE offers an interactive dashboard with multiple functionalities, including platform and study overviews, filters, visualization plots, and report generation. Additionally, it can be embedded via iframe within partner platform websites, with optional user authentication. A metadata submission tool was developed separately to encourage data custodians to contribute new datasets.

**Result:**

GLADSE centralizes metadata exploration, enabling users to identify and compare relevant datasets without navigating multiple platforms. Its modular design allows seamless updates and future expansions. The tool improves data accessibility by featuring diverse metadata and presenting it in an interactive interface. Early feedback highlights its effectiveness in improving data discovery and enabling cross‐platform collaboration, including federated analysis. By integrating global resources, the tool maximizes the utility of existing research infrastructure and facilitates global partnerships.

**Conclusion:**

GLADSE advances dementia research making data FAIR and fostering collaboration across dementia platforms. Planned future developments, such as incorporating measurement‐level metadata standardised using the C‐SURV ontology will enhance the utility of study filters and visualization plots. Expanding to include detailed imaging and genomic metadata will further strengthen its utility. By proposing a solution to the decentralized data platforms and promoting global collaboration, GLADSE maximizes the value of public investments in research infrastructures, supports critical progress in understanding, preventing, and treating dementia, a pressing public health challenge.

